# The Role of Leucine-Rich Repeat Containing Protein 10 (LRRC10) in Dilated Cardiomyopathy

**DOI:** 10.3389/fphys.2016.00337

**Published:** 2016-08-03

**Authors:** Matthew J. Brody, Youngsook Lee

**Affiliations:** ^1^Department of Pediatrics, Cincinnati Children's Hospital Medical CenterCincinnati, OH, USA; ^2^Department of Cell and Regenerative Biology, University of Wisconsin-MadisonMadison, WI, USA

**Keywords:** LRRC10, leucine-rich repeat, dilated cardiomyopathy, cardiomyopathy, eccentric hypertrophy

## Abstract

Leucine-rich repeat containing protein 10 (LRRC10) is a cardiomyocyte-specific member of the Leucine-rich repeat containing (LRRC) protein superfamily with critical roles in cardiac function and disease pathogenesis. Recent studies have identified *LRRC10* mutations in human idiopathic dilated cardiomyopathy (DCM) and *Lrrc10* homozygous knockout mice develop DCM, strongly linking LRRC10 to the molecular etiology of DCM. LRRC10 localizes to the dyad region in cardiomyocytes where it can interact with actin and α-actinin at the Z-disc and associate with T-tubule components. Indeed, this region is becoming increasingly recognized as a signaling center in cardiomyocytes, not only for calcium cycling, excitation-contraction coupling, and calcium-sensitive hypertrophic signaling, but also as a nodal signaling hub where the myocyte can sense and respond to mechanical stress. Disruption of a wide range of critical structural and signaling molecules in cardiomyocytes confers susceptibility to cardiomyopathies in addition to the more classically studied mutations in sarcomeric proteins. However, the molecular mechanisms underlying DCM remain unclear. Here, we review what is known about the cardiomyocyte functions of LRRC10, lessons learned about LRRC10 and DCM from the *Lrrc10* knockout mouse model, and discuss ongoing efforts to elucidate molecular mechanisms whereby mutation or absence of LRRC10 mediates cardiac disease.

## Introduction

Dilated cardiomyopathy (DCM) and hypertrophic cardiomyopathy (HCM) are the most common primary myocardial diseases with a prevalence of at least 1 in 2500 and 1 in 500 individuals, respectively, (McNally et al., 2013, 2015; Kimura, 2016). DCM is characterized by eccentric cardiac growth resulting in ventricular dilation and reduced cardiac function without an increase in ventricular wall thickness (Maillet et al., 2013; van Berlo et al., 2013; Bang, 2016; Kimura, 2016). In contrast, in HCM, the heart undergoes concentric growth that results in ventricular wall thickening and reduced ventricular inner diameter, ultimately resulting in reduced cardiac output (Maillet et al., 2013; van Berlo et al., 2013; Bang, 2016). Both HCM and DCM can progress to congestive heart failure and are associated with an increased risk of sudden death (Maillet et al., 2013; van Berlo et al., 2013). While a number of mutations of genes encoding sarcomeric proteins are known to cause HCM, the genetic etiology of DCM is much more heterogeneous, including mutation of genes encoding proteins of the Z-disc, costamere, cytoskeleton, sarcolemma, sarcomere, and nuclear lamina (Cheng et al., 2010; McNally et al., 2013, 2015; Bang, 2016). The most prevalent DCM causing mutations are truncations of the sarcomeric protein titin (Herman et al., 2012; Hinson et al., 2015). DCM can also occur in response to myocardial infarction or ischemic damage, which accounts for about half of all cases, while the majority of remaining cases are idiopathic, underscoring the need to identify more genetic mutations that underlie DCM (McNally et al., 2013). The genetic determinants and molecular etiology underlying DCM in a majority of patients remain unclear.

Leucine-rich repeat containing protein 10 (LRRC10) was identified based on its cardiac-specific expression pattern (Nakane et al., 2004; Adameyko et al., 2005; Kim et al., 2007b). LRRC10 is highly conserved (Kim et al., 2007a) and exclusively expressed in cardiomyocytes (Kim et al., 2007b; Brody et al., 2013), suggesting critical cardiac functions for LRRC10. Recently, studies in *Lrrc10* knockout mice (Brody et al., 2012, 2016) and the identification of LRRC10 mutations in human DCM (Qu et al., 2015) have sparked interest in the underlying molecular mechanisms that mediate cardiac disease when LRRC10 is absent or mutated. LRRC10 belongs to the diverse LRRC protein superfamily, which is comprised of many proteins that have in common their leucine-rich repeat (LRR) domains that function as protein interaction motifs (Kobe and Deisenhofer, 1994, 1995; Kobe and Kajava, 2001). LRRs are sequences of 20–30 amino acids rich in leucine and other aliphatic amino acids. LRRCs contain two or more LRRs aligned in tandem to form a curved non-globular, solenoid-shaped structure that is ideal for mediating protein:protein interactions (Kobe and Deisenhofer, 1994, 1995; Bella et al., 2008). LRRC10 is about 32 kDa, and has no known functional domains except its seven LRRs (Kim et al., 2007a,b), suggesting that its molecular functions rely on protein interactions.

LRRC10 is expressed in the developing heart and upregulated at birth with elevated protein levels maintained in adulthood (Brody et al., 2013). Cardiomyocyte-specific expression of LRRC10 is tightly controlled by the cardiac transcription factors Nkx2-5, GATA4, and serum response factor (SRF) via conserved regulatory elements near the LRRC10 promoter region (Fan et al., 2011; Brody et al., 2013). Investigation of *Lrrc10* homozygous knockout (*Lrrc10*^−∕−^) mice (Manuylov et al., 2008) has led to recent discoveries linking LRRC10 to the molecular etiology of DCM (Brody et al., 2012; Qu et al., 2015). Here, we review recent findings in the *Lrrc10*^−∕−^ mouse and human idiopathic DCM patients that implicate LRRC10 in the pathogenesis of DCM, discuss molecular alterations in the *Lrrc10*^−∕−^ heart that may contribute to cardiomyopathy, and preface ongoing work investigating the molecular function of LRRC10.

## LRRC10 deletion causes dilated cardiomyopathy in mice

**Figure 1:**
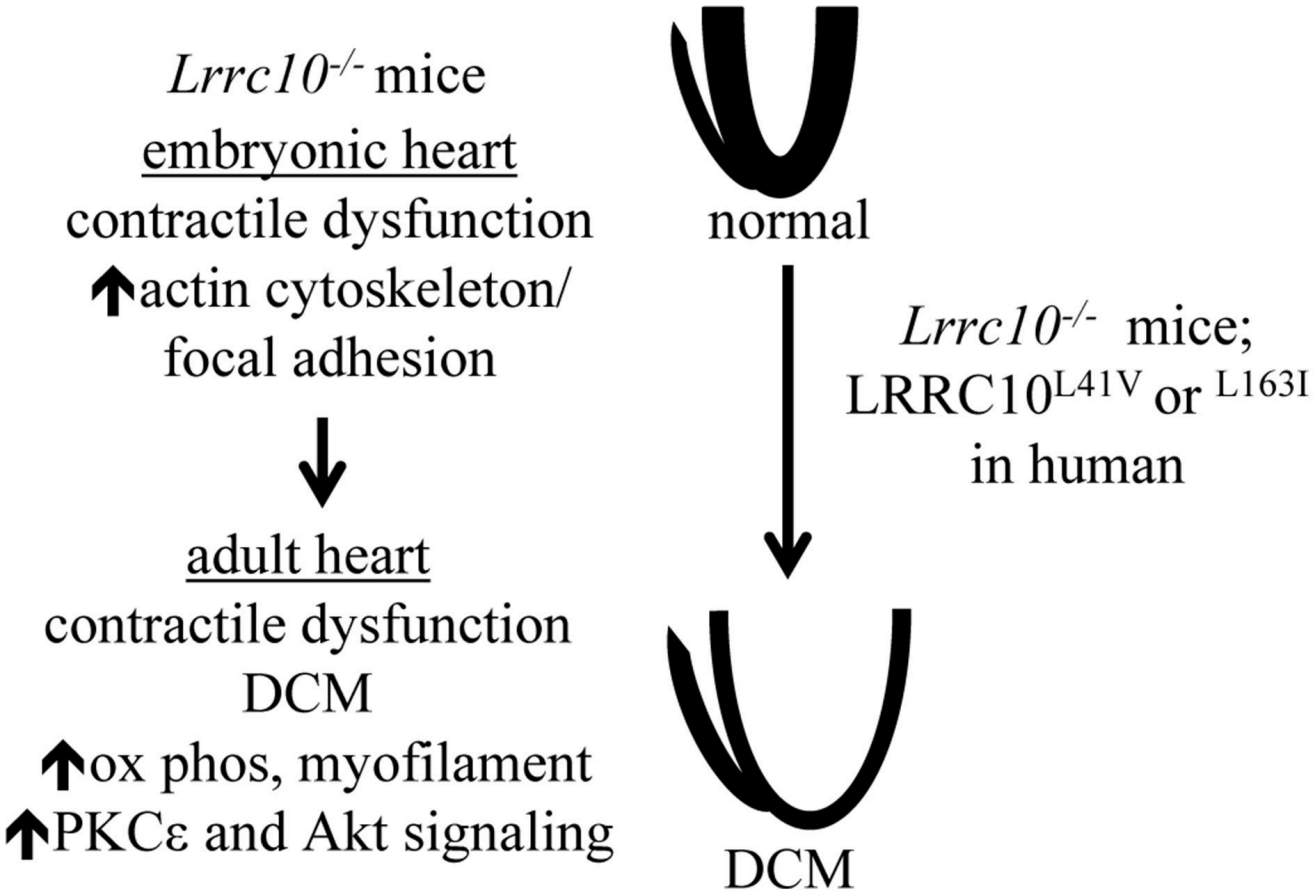
**Molecular and functional alterations in ***Lrrc10***^**−∕−**^ mice**. *Lrrc10*^−∕−^ mice have reduced cardiac contractility prior to birth that is associated with the upregulation of actin cytoskeletal transcripts and proteins. Perinatal cardiac dysfunction in *Lrrc10*^−∕−^ mice progresses to DCM in adulthood and is associated with upregulation of transcripts involved in oxidative phosphorylation (ox phos) and myofilament contraction and activation of Akt and PKCε signaling. L41V or L163I mutations in LRRC10 cause DCM in humans.

Pioneering studies in zebrafish demonstrated that Lrrc10 is required for normal cardiac function in vertebrates (Kim et al., 2007a). Knockdown of Lrrc10 in zebrafish causes cardiac developmental defects, reduced cardiac function, and lethality (Kim et al., 2007a). To investigate LRRC10 function in the mammalian heart, *Lrrc10*^−∕−^ mice were generated (Manuylov et al., 2008). *Lrrc10*^−∕−^ mice exhibit perinatal cardiomyopathy and progressive DCM in adulthood (Brody et al., 2012). *Lrrc10*^−∕−^ mice have reduced cardiac function prior to birth that progresses to eccentric cardiac growth, ventricular dilation, and further deterioration of cardiac function in adult mice (Brody et al., 2012; Figure 1). These studies established the *Lrrc10*^−∕−^ mouse as a novel model of pediatric cardiomyopathy and implicated LRRC10 as a candidate DCM gene in humans. Moreover, *Lrrc10*^−∕−^ mice exhibit greatly reduced cardiac contractility and exacerbated remodeling in response to pressure overload induced by transverse aortic constriction (Brody et al., 2016). The accelerated progression of DCM observed in *Lrrc10*^−∕−^ mice after pressure overload indicates that deletion of LRRC10 renders the heart sensitive to disease pathogenesis during hypertensive remodeling, suggesting that human patients with mutations in the *LRRC10* gene may be prone to more fulminant disease and DCM under conditions of pressure overload, such as aortic stenosis or elevated blood pressure.

*Lrrc10*^−∕−^ mice exhibit an uncommon form of cardiac remodeling characterized by direct progression to ventricular dilation without compensatory concentric hypertrophic growth, and cardiac functional impairment in the absence of an increase in myocyte death or cardiac fibrosis (Brody et al., 2012). In response to pressure overload *Lrrc10*^−∕−^ mice are capable of mounting an appropriate concentric cardiac hypertrophy response, which is accompanied by further eccentric cardiac growth and dilation with dramatically reduced cardiac functional performance but similar fibrotic remodeling and cardiomyocyte death compared to controls (Brody et al., 2016). Thus, LRRC10 appears to be required to maintain cardiac contractile function and its absence causes dilative cardiac remodeling.

Analyses of adult *Lrrc10*^−∕−^ hearts identified transcriptional and molecular alterations during the progression of DCM. Pathway analysis of gene expression profiling in adult *Lrrc10*^−∕−^ hearts revealed upregulation of genes involved in oxidative phosphorylation and myofilament contraction as the most prominent transcriptional alterations, including cytochrome c oxidase, ATP synthase, and NADH dehydrogenase genes, and *Tnni3, Tnnt1, Tpm1*, and *Mybpc3* (Brody et al., 2012; Figure 1). Despite increased transcript levels, myofilament protein abundance was not increased in *Lrrc10*^−∕−^ hearts (Brody et al., 2012), likely because the ordered myofilament lattice contains stoichiometric quantities of sarcomeric proteins (Michele et al., 1999) and thus cannot accommodate additional myofilament proteins beyond the normal rate of turnover, even if transcript levels are elevated. Upregulation of transcripts involved in oxidative phosphorylation and myofilament contraction in the *Lrrc10*^−∕−^ heart are likely a compensatory attempt to bolster cardiac bioenergetics and sarcomeric proteins, respectively, to cope with diminished contractile function.

Various signaling pathways play important roles in regulating cardiac function and the progression to cardiomyopathy and heart failure (McNally et al., 2013; van Berlo et al., 2013). However, precise roles of specific signaling pathways in the progression of DCM have not been fully elucidated. *Lrrc10*^−∕−^ hearts activate protein kinase C ε (PKCε) and Akt signaling (Brody et al., 2012; Figure 1). PKCε is activated downstream of GPCR agonist or mechanical stimulation and is thought to be cardioprotective in large part due to its augmentation of mitochondrial function (Inagaki et al., 2003; Iwata et al., 2005; McCarthy et al., 2005; Budas and Mochly-Rosen, 2007). Activation of PKCε reduces ventricular dilation and hypertrophy but does not rescue contractile dysfunction in the cTnT^R141W^ transgenic mouse model of DCM (Lu et al., 2014), suggesting that PKCε may partially ameliorate pathological cardiac remodeling in some forms of DCM. In this regard, activation of PKCε potentially prevents the progression to congestive heart failure in *Lrrc10*^−∕−^ mice.

Akt is a serine/threonine kinase that is protective in the heart predominantly due to its antiapoptotic effects on myocytes (Miyamoto et al., 2009). Adult *Lrrc10*^−∕−^ hearts exhibit elevated levels of active Akt phosphorylated at Ser-473 (Brody et al., 2012), which may limit cardiomyocyte apoptosis. Activation of Akt and upregulation of oxidative phosphorylation and myofilament contraction genes observed in *Lrrc10*^−∕−^ hearts have also been reported in human DCM and heart failure (Haq et al., 2001; Barrans et al., 2002; Grzeskowiak et al., 2003; Asakura and Kitakaze, 2009; Colak et al., 2009; Sopko et al., 2011). Therefore, these molecular alterations may represent compensatory protective pathways associated with the progression of DCM. However, it remains unknown how these signaling pathways are upregulated and what the downstream effects are in the *Lrrc10*^−∕−^ heart. It is plausible that PKCε or Akt has novel roles regulating downstream signaling pathways that function in the setting of cardiac dysfunction and remodeling caused by LRRC10 deletion. Thus, it would be interesting to determine whether upregulation of these pathways is indeed cardioprotective in certain forms of DCM, which would aid in the identification of therapeutic targets for the treatment of heart failure patients.

To investigate potential pathogenic mechanisms underlying DCM caused by LRRC10 deficiency, molecular alterations were identified in embryonic *Lrrc10*^−∕−^ hearts (Brody et al., 2012). Gene expression profiling of *Lrrc10*^−∕−^ hearts at embryonic day (E) 15.5, prior to the development of cardiac dysfunction at E17.5 or eccentric ventricular remodeling that occurs in adulthood, revealed upregulation of the actin cytoskeleton and focal adhesion gene pathways to be the most significantly dysregulated gene networks (Brody et al., 2012; Figure 1). Embryonic *Lrrc10*^−∕−^ hearts have elevated transcripts for integrin β, integrin-linked kinase (ILK), and parvin-α, which are all induced in the heart in response to biomechanical stress (Babbitt et al., 2002; Zemljic-Harpf et al., 2007; Sopko et al., 2011). Upregulation of integrin β1, vinculin, and talin was observed at the protein level in embryonic *Lrrc10*^−∕−^ hearts (Brody et al., 2012), suggesting alterations in focal adhesion complexes and a potential mechanosensing role for LRRC10 (See Discussion section below).

## Molecular functions of LRRC10

LRRC10 was shown to interact with α-actinin and α-sarcomeric actin at the Z-disc in cardiomyocytes and directly bind all actin isoforms (Brody et al., 2012). Interaction of LRRC10 with actin thin filaments appears to be dynamic as this interaction is reduced in response to pressure overload (Brody et al., 2016). Thus, LRRC10 may localize to the Z-disc, T-tubule, or other locations within the cardiomyocyte in a stimulus-dependent manner or in response to certain mechanical or molecular signals, potentially using its interaction with actin at the Z-disc or cytoskeleton as a docking station.

Transmission electron microscopy analyses demonstrated that LRRC10 localizes to the dyad region in cardiomyocytes (Kim et al., 2007b), where the T-tubule comes into close juxtaposition to the Z-disc and sarcoplasmic reticulum (SR). The dyad serves as a critical link between the T-tubule network, SR, Z-disc and cytoskeletal proteins, with roles in regulating and anchoring ion channels, contractile and structural proteins, and signaling molecules. Localization of LRRC10 near the Z-disc positions it at an optimal subcellular location to mediate signaling responses to mechanical stress (Frank and Frey, 2011). The Z-disc contains mechanical scaffolding and signaling molecules that structurally and functionally link the myofilament to the costamere, cytoskeleton, and extracellular matrix (Ervasti, 2003; Luther, 2009; Frank and Frey, 2011). Genetic deletion or mutation of many genes encoding Z-disc and cytoskeletal proteins results in DCM in mice and humans, including Cypher (Vatta et al., 2003; Zheng et al., 2009), muscle LIM protein (MLP) (Arber et al., 1997; Knoll et al., 2002), integrin-linked kinase (ILK) (White et al., 2006; Knoll et al., 2007), vinculin (Zemljic-Harpf et al., 2007), and desmin (Li et al., 1999). Thus, defects in focal adhesion complex, cytoskeletal, or Z-disc components can participate in the pathobiology of DCM. MLP anchors calcineurin at the Z-disc to mediate activation of downstream NFAT-dependent prohypertrophic gene expression in response to myocardial infarction (Heineke et al., 2005) and also shuttles between the Z-disc and nucleus in response to certain stimuli (Ecarnot-Laubriet et al., 2000; Boateng et al., 2007, 2009). Therefore, LRRC10 may bind actin to properly localize, displace, or regulate the function of an interacting factor at the Z-disc or may dissociate from the Z-disc to other subcellular locations to perform regulatory functions.

Other LRRCs bind actin, suggesting that actin binding may be a general mechanism for LRRCs to localize or dock at specific cellular locations. For example, LRRC67 binds all actin isoforms (Wang et al., 2010) and the LRR domain of tropomodulin-1 mediates its binding to sarcomeric actin in cardiomyocytes (Tsukada et al., 2011). LRRCs have also been previously reported to have mechanosensing functions. The striated-muscle-specific protein, LRRC39, localizes to the M-line in cardiomyocytes and regulates SRF-dependent transcription (Will et al., 2010), suggesting that LRRCs may serve as local mechanosensors in cardiomyocytes.

*Lrrc10*^−∕−^ mice exhibit defective cardiac contractility prior to ventricular remodeling (Brody et al., 2012), and severely compromised cardiac function in response to pressure overload (Brody et al., 2016), indicating that LRRC10 is necessary to maintain cardiac contractile function. Nonetheless, LRRC10 does not have a role in directly regulating cardiomyocyte contraction at the level of the myofilament (Brody et al., 2012, 2016). No alterations in force development or myofilament calcium sensitivity were detected in skinned myocardium from *Lrrc10*^−∕−^ hearts (Brody et al., 2012). Although single cell contractility is not altered at baseline in isolated *Lrrc10*^−∕−^ myocytes, the contractile response to β-adrenergic stimulation is blunted (Brody et al., 2016). These data indicate that LRRC10 does not directly regulate myofilament contraction or cross-bridge cycling. This is consistent with a mechanosensing function for LRRC10. In the loaded, intact heart where cardiomyocytes must sense mechanical stress and preload, LRRC10 is required for cardiomyocyte contractile function. In contrast, in unloaded isolated myocytes where mechanical strain cannot be sensed by the myocyte, LRRC10 is dispensable for cardiomyocyte contractility.

These data are also consistent with a role for LRRC10 in excitation-contraction coupling such that when LRRC10 is absent or mutated, calcium cycling is defective resulting in aberrant levels of calcium available at the myofilament to stimulate contraction (Luo and Anderson, 2013). Although the reduced contractile response of isolated *Lrrc10*^−∕−^ myocytes to β-adrenergic stimulation (Brody et al., 2016) could be a consequence of DCM in *Lrrc10*^−∕−^ mice, it could also be explained by a primary function for LRRC10 in coupling adrenergic stimulation to myofilament contraction by facilitating excitation-contraction coupling.

It is intriguing that LRRCs have been shown to regulate ion channel activity. Activation of the large-conductance, calcium- and voltage-activated potassium (BK) channel (encoded by the *Slo1* gene) is regulated by LRRC26, which serves as an auxiliary γ-subunit to regulate BK channel activity (Braun, 2010; Yan and Aldrich, 2010; Evanson et al., 2014). Moreover, LRRC52 acts as a testis-specific γ-subunit regulator of the alkalization-activated Slo3 potassium channel (Yang et al., 2011), indicating not only that some LRRCs may function as regulators of the Slo family of potassium channels (Zhang and Yan, 2014), but also that LRRCs may provide tissue specificity to the regulation of ion channel activity. Thus, LRRC10 may serve as a cardiomyocyte-specific auxiliary protein and regulator of ion channel activity to mediate appropriate excitation-contraction coupling and resultant contractility in cardiomyocytes.

## Association of LRRC10 mutations with dilated cardiomyopathy in humans

Two heterozygous mutations in *LRRC10* were identified in human patients with idiopathic DCM (Qu et al., 2015). These studies identified p.L41V and p.L163I missense mutations in LRRC10 in two unrelated families with DCM. Both mutations were inherited in an autosomal dominant manner and co-segregated with DCM with complete penetrance (Qu et al., 2015). Residues L41 and L163 are highly conserved, suggesting they are critical for the structure and/or function of the LRRC10 protein. These mutants may not localize properly to the Z-disc or T-tubule in the dyad region. Alternatively, LRRC10 DCM-associated mutants may lose their ability to physically or functionally interact with cofactors. The amino acid substitutions in these LRRC10 mutants (L41V and L163I) are relatively moderate biochemical alterations and not predicted to drastically alter the overall three dimensional conformation of the LRRC10 protein. Thus, it remains unknown if the L41V or L163I mutants function as a dominant negative or a loss- or gain-of-function mutation. Further investigation is necessary to determine precisely how these mutations alter the molecular structure of LRRC10 and how this mechanistically perturbs LRRC10 function to cause disease.

Future sequencing for *LRRC10* in human idiopathic DCM will be very informative on the prevalence of *LRRC10* mutations in DCM and the potential for additional mutations to contribute to human cardiac disease. Identification of additional pathogenic mutations in the LRRC10 protein coupled with molecular studies of recently identified LRRC10 disease-associated mutants will shed light on how these mutations disrupt LRRC10 function and cause DCM.

## Perspectives

Much insight has been gained into the role of LRRC10 in DCM and molecular mechanisms of DCM pathogenesis in recent years. The identification of two novel mutations in LRRC10 that are associated with human idiopathic DCM has opened the door for investigation into the roles of LRRC10 in human cardiac disease and the underlying molecular mechanisms that cause DCM in response to mutation or genetic ablation of LRRC10. The *Lrrc10*^−∕−^ mouse has provided a valuable animal model to investigate the molecular function of LRRC10 that can translate to understanding of human disease. Molecular evidence and investigation of *Lrrc10*^−∕−^ mice thus far has pointed to roles for LRRC10 in mechanosensing and/or excitation-contraction coupling. Future studies will investigate calcium cycling in *Lrrc10*^−∕−^ cardiomyocytes to determine if LRRC10 has a fundamental role in regulation of excitation-contraction coupling. Identification of proteins that functionally interact with LRRC10 will be crucial to determine the molecular functions of LRRC10 and mechanistic basis of DCM. Further sequencing of LRRC10 in human idiopathic DCM will reveal the prevalence of LRRC10 mutations in DCM and potentially identify novel mutations associated with cardiac disease in humans. Generation of knock-in mice for human DCM associated LRRC10 mutations will provide valuable models to investigate molecular mechanisms of DCM. Moreover, generation of cardiomyocytes from patient-derived induced pluripotent stem cells (iPSCs) or human pluripotent stem cells (Sharma et al., 2013) containing DCM-linked LRRC10 mutations will serve as powerful tools to investigate human cardiomyopathy. These studies will provide models to test therapeutic strategies to treat DCM and aid in the discovery of pathogenic mechanisms underlying DCM in human patients.

## Author contributions

MB and YL made substantial contributions to the conception and design of the manuscript, drafted and critically revised the manuscript, approved the final version of the manuscript, and agree to be accountable for all aspects of the work.

## Funding

This work was supported by National Institutes of Health (NIH) Grant HL-067050 and American Heart Association (AHA) Grant 12GRNT12070021 (to YL), AHA Predoctoral Fellowship 11PRE5580012 (to MB), and NIH Molecular and Environmental Toxicology Training Grant T32ES007015.

### Conflict of interest statement

The authors declare that the research was conducted in the absence of any commercial or financial relationships that could be construed as a potential conflict of interest.

## References

[B1] Adameyko I. I. Mudry R. E. Houston-Cummings N. R. Veselov A. P. Gregorio C. C. Tevosian S. G. (2005). Expression and regulation of mouse SERDIN1, a highly conserved cardiac-specific leucine-rich repeat protein. Dev. Dyn. 233, 540–552. 10.1002/dvdy.20368 15830381

[B2] Arber S. Hunter J. J. Ross J. Jr. Hongo M. Sansig G. Borg J.. (1997). MLP-deficient mice exhibit a disruption of cardiac cytoarchitectural organization, dilated cardiomyopathy, and heart failure. Cell 88, 393–403. 10.1016/S0092-8674(00)81878-4 9039266

[B3] Asakura M. Kitakaze M. (2009). Global gene expression profiling in the failing myocardium. Circ. J. 73, 1568–1576. 10.1253/circj.CJ-09-0465 19638707

[B4] Babbitt C. J. Shai S. Y. Harpf A. E. Pham C. G. Ross R. S. (2002). Modulation of integrins and integrin signaling molecules in the pressure-loaded murine ventricle. Histochem. Cell Biol. 118, 431–439. 10.1007/s00418-002-0476-1 12483308

[B5] Bang M. L. (2016). Animal models of congenital cardiomyopathies associated with mutations in Z-line proteins. J. Cell. Physiol.. [Epub ahead of print]. 10.1002/jcp.25424 27171814

[B6] Barrans J. D. Allen P. D. Stamatiou D. Dzau V. J. Liew C. C. (2002). Global gene expression profiling of end-stage dilated cardiomyopathy using a human cardiovascular-based cDNA microarray. Am. J. Pathol. 160, 2035–2043. 10.1016/S0002-9440(10)61153-4 12057908 PMC1850841

[B7] Bella J. Hindle K. L. McEwan P. A. Lovell S. C. (2008). The leucine-rich repeat structure. Cell. Mol. Life Sci. 65, 2307–2333. 10.1007/s00018-008-8019-0 18408889 PMC11131621

[B8] Boateng S. Y. Belin R. J. Geenen D. L. Margulies K. B. Martin J. L. Hoshijima M.. (2007). Cardiac dysfunction and heart failure are associated with abnormalities in the subcellular distribution and amounts of oligomeric muscle LIM protein. Am. J. Physiol. Heart Circ. Physiol. 292, H259–H269. 10.1152/ajpheart.00766.2006 16963613

[B9] Boateng S. Y. Senyo S. E. Qi L. Goldspink P. H. Russell B. (2009). Myocyte remodeling in response to hypertrophic stimuli requires nucleocytoplasmic shuttling of muscle LIM protein. J. Mol. Cell. Cardiol. 47, 426–435. 10.1016/j.yjmcc.2009.04.006 19376126 PMC2739242

[B10] Braun A. P. (2010). A new “opening” act on the BK channel stage: identification of LRRC26 as a novel BK channel accessory subunit that enhances voltage-dependent gating. Channels 4, 249–250. 10.4161/chan.4.4.13330 21057223

[B11] Brody M. J. Cho E. Mysliwiec M. R. Kim T. G. Carlson C. D. Lee K. H.. (2013). Lrrc10 is a novel cardiac-specific target gene of Nkx2-5 and GATA4. J. Mol. Cell. Cardiol. 62, 237–246. 10.1016/j.yjmcc.2013.05.020 23751912 PMC3940241

[B12] Brody M. J. Feng L. Grimes A. C. Hacker T. A. Olson T. M. Kamp T. J.. (2016). LRRC10 is required to maintain cardiac function in response to pressure overload. Am. J. Physiol. Heart Circ. Physiol. 310, H269–H278. 10.1152/ajpheart.00717.2014 26608339 PMC4747898

[B13] Brody M. J. Hacker T. A. Patel J. R. Feng L. Sadoshima J. Tevosian S. G.. (2012). Ablation of the cardiac-specific gene leucine-rich repeat containing 10 (lrrc10) results in dilated cardiomyopathy. PLoS ONE 7:e51621. 10.1371/journal.pone.0051621 23236519 PMC3517560

[B14] Budas G. R. Mochly-Rosen D. (2007). Mitochondrial protein kinase Cepsilon (PKCepsilon): emerging role in cardiac protection from ischaemic damage. Biochem. Soc. Trans. 35(Pt 5), 1052–1054. 10.1042/BST0351052 17956277

[B15] Cheng H. Kimura K. Peter A. K. Cui L. Ouyang K. Shen T.. (2010). Loss of enigma homolog protein results in dilated cardiomyopathy. Circ. Res. 107, 348–356. 10.1161/CIRCRESAHA.110.218735 20538684 PMC3684396

[B16] Colak D. Kaya N. Al-Zahrani J. Al Bakheet A. Muiya P. Andres E.. (2009). Left ventricular global transcriptional profiling in human end-stage dilated cardiomyopathy. Genomics 94, 20–31. 10.1016/j.ygeno.2009.03.003 19332114 PMC4152850

[B17] Ecarnot-Laubriet A. De Luca K. Vandroux D. Moisant M. Bernard C. Assem M.. (2000). Downregulation and nuclear relocation of MLP during the progression of right ventricular hypertrophy induced by chronic pressure overload. J. Mol. Cell. Cardiol. 32, 2385–2395. 10.1006/jmcc.2000.1269 11113014

[B18] Ervasti J. M. (2003). Costameres: the Achilles' heel of Herculean muscle. J. Biol. Chem. 278, 13591–13594. 10.1074/jbc.R200021200 12556452

[B19] Evanson K. W. Bannister J. P. Leo M. D. Jaggar J. H. (2014). LRRC26 is a functional BK channel auxiliary gamma subunit in arterial smooth muscle cells. Circ. Res. 115, 423–431. 10.1161/CIRCRESAHA.115.303407 24906643 PMC4119551

[B20] Fan X. Yang Q. Wang Y. Zhang Y. Wang J. Yuan J.. (2011). Cloning and characterization of the cardiac-specific Lrrc10 promoter. BMB Rep. 44, 123–128. 10.5483/BMBRep.2011.44.2.123 21345312

[B21] Frank D. Frey N. (2011). Cardiac Z-disc Signaling Network. J. Biol. Chem. 286, 9897–9904. 10.1074/jbc.R110.174268 21257757 PMC3060542

[B22] Grzeskowiak R. Witt H. Drungowski M. Thermann R. Hennig S. Perrot A.. (2003). Expression profiling of human idiopathic dilated cardiomyopathy. Cardiovasc. Res. 59, 400–411. 10.1016/S0008-6363(03)00426-7 12909323

[B23] Haq S. Choukroun G. Lim H. Tymitz K. M. del Monte F. Gwathmey J.. (2001). Differential activation of signal transduction pathways in human hearts with hypertrophy versus advanced heart failure. Circulation 103, 670–677. 10.1161/01.CIR.103.5.670 11156878

[B24] Heineke J. Ruetten H. Willenbockel C. Gross S. C. Naguib M. Schaefer A.. (2005). Attenuation of cardiac remodeling after myocardial infarction by muscle LIM protein-calcineurin signaling at the sarcomeric Z-disc. Proc. Natl. Acad. Sci. U.S.A. 102, 1655–1660. 10.1073/pnas.0405488102 15665106 PMC547821

[B25] Herman D. S. Lam L. Taylor M. R. Wang L. Teekakirikul P. Christodoulou D.. (2012). Truncations of titin causing dilated cardiomyopathy. N. Engl. J. Med. 366, 619–628. 10.1056/NEJMoa1110186 22335739 PMC3660031

[B26] Hinson J. T. Chopra A. Nafissi N. Polacheck W. J. Benson C. C. Swist S.. (2015). HEART DISEASE. Titin mutations in iPS cells define sarcomere insufficiency as a cause of dilated cardiomyopathy. .Science 349, 982–986. 10.1126/science.aaa5458 26315439 PMC4618316

[B27] Inagaki K. Hahn H. S. Dorn G. W. II. Mochly-Rosen D. (2003). Additive protection of the ischemic heart *ex vivo* by combined treatment with delta-protein kinase C inhibitor and epsilon-protein kinase C activator. Circulation 108, 869–875. 10.1161/01.CIR.0000081943.93653.73 12860903

[B28] Iwata M. Maturana A. Hoshijima M. Tatematsu K. Okajima T. Vandenheede J. R.. (2005). PKCepsilon-PKD1 signaling complex at Z-discs plays a pivotal role in the cardiac hypertrophy induced by G-protein coupling receptor agonists. Biochem. Biophys. Res. Commun. 327, 1105–1113. 10.1016/j.bbrc.2004.12.128 15652511 PMC3224855

[B29] Kim K. H. Antkiewicz D. S. Yan L. Eliceiri K. W. Heideman W. Peterson R. E.. (2007a). Lrrc10 is required for early heart development and function in zebrafish. Dev. Biol. 308, 494–506. 10.1016/j.ydbio.2007.06.005 17601532 PMC2048587

[B30] Kim K. H. Kim T. G. Micales B. K. Lyons G. E. Lee Y. (2007b). Dynamic expression patterns of leucine-rich repeat containing protein 10 in the heart. Dev. Dyn. 236, 2225–2234. 10.1002/dvdy.21225 17626279 PMC2002521

[B31] Kimura A. (2016). Molecular genetics and pathogenesis of cardiomyopathy. J. Hum. Genet. 61, 41–50. 10.1038/jhg.2015.83 26178429

[B32] Knoll R. Hoshijima M. Hoffman H. M. Person V. Lorenzen-Schmidt I. Bang M. L.. (2002). The cardiac mechanical stretch sensor machinery involves a Z disc complex that is defective in a subset of human dilated cardiomyopathy. Cell 111, 943–955. 10.1016/S0092-8674(02)01226-6 12507422

[B33] Knoll R. Postel R. Wang J. Kratzner R. Hennecke G. Vacaru A. M.. (2007). Laminin-alpha4 and integrin-linked kinase mutations cause human cardiomyopathy via simultaneous defects in cardiomyocytes and endothelial cells. Circulation 116, 515–525. 10.1161/CIRCULATIONAHA.107.689984 17646580

[B34] Kobe B. Deisenhofer J. (1994). The leucine-rich repeat: a versatile binding motif. Trends Biochem. Sci. 19, 415–421. 10.1016/0968-0004(94)90090-6 7817399

[B35] Kobe B. Deisenhofer J. (1995). A structural basis of the interactions between leucine-rich repeats and protein ligands. Nature 374, 183–186. 7877692 10.1038/374183a0

[B36] Kobe B. Kajava A. V. (2001). The leucine-rich repeat as a protein recognition motif. Curr. Opin. Struct. Biol. 11, 725–732. 10.1016/S0959-440X(01)00266-4 11751054

[B37] Li D. Tapscoft T. Gonzalez O. Burch P. E. Quinones M. A. Zoghbi W. A.. (1999). Desmin mutation responsible for idiopathic dilated cardiomyopathy. Circulation 100, 461–464. 10.1161/01.CIR.100.5.461 10430757

[B38] Lu D. Zhang L. Bao D. Lu Y. Zhang X. Liu N.. (2014). Calponin1 inhibits dilated cardiomyopathy development in mice through the epsilonPKC pathway. Int. J. Cardiol. 173, 146–153. 10.1016/j.ijcard.2014.02.032 24631115

[B39] Luo M. Anderson M. E. (2013). Mechanisms of altered Ca(2)(+) handling in heart failure. Circ. Res. 113, 690–708. 10.1161/CIRCRESAHA.113.301651 23989713 PMC4080816

[B40] Luther P. K. (2009). The vertebrate muscle Z-disc: sarcomere anchor for structure and signalling. J. Muscle Res. Cell Motil. 30, 171–185. 10.1007/s10974-009-9189-6 19830582 PMC2799012

[B41] Maillet M. van Berlo J. H. Molkentin J. D. (2013). Molecular basis of physiological heart growth: fundamental concepts and new players. Nat. Rev. Mol. Cell Biol. 14, 38–48. 10.1038/nrm3495 23258295 PMC4416212

[B42] Manuylov N. L. Manuylova E. Avdoshina V. Tevosian S. (2008). Serdin1/Lrrc10 is dispensable for mouse development. Genesis 46, 441–446. 10.1002/dvg.20422 18781631

[B43] McCarthy J. McLeod C. J. Minners J. Essop M. F. Ping P. Sack M. N. (2005). PKCepsilon activation augments cardiac mitochondrial respiratory post-anoxic reserve–a putative mechanism in PKCepsilon cardioprotection. J. Mol. Cell. Cardiol. 38, 697–700. 10.1016/j.yjmcc.2005.02.010 15808847

[B44] McNally E. M. Barefield D. Y. Puckelwartz M. J. (2015). The genetic landscape of cardiomyopathy and its role in heart failure. Cell Metab. 21, 174–182. 10.1016/j.cmet.2015.01.013 25651172 PMC4331062

[B45] McNally E. M. Golbus J. R. Puckelwartz M. J. (2013). Genetic mutations and mechanisms in dilated cardiomyopathy. J. Clin. Invest. 123, 19–26. 10.1172/JCI62862 23281406 PMC3533274

[B46] Michele D. E. Albayya F. P. Metzger J. M. (1999). Thin filament protein dynamics in fully differentiated adult cardiac myocytes: toward a model of sarcomere maintenance. J. Cell Biol. 145, 1483–1495. 10.1083/jcb.145.7.1483 10385527 PMC2133172

[B47] Miyamoto S. Rubio M. Sussman M. A. (2009). Nuclear and mitochondrial signalling Akts in cardiomyocytes. Cardiovasc. Res. 82, 272–285. 10.1093/cvr/cvp087 19279164 PMC2675933

[B48] Nakane T. Satoh T. Inada Y. Nakayama J. Itoh F. Chiba S. (2004). Molecular cloning and expression of HRLRRP, a novel heart-restricted leucine-rich repeat protein. Biochem. Biophys. Res. Commun. 314, 1086–1092. 10.1016/j.bbrc.2003.12.202 14751244

[B49] Qu X. K. Yuan F. Li R. G. Xu L. Jing W. F. Liu H.. (2015). Prevalence and spectrum of LRRC10 mutations associated with idiopathic dilated cardiomyopathy. Mol. Med. Rep. 12, 3718–3724. 10.3892/mmr.2015.3843 26017719

[B50] Sharma A. Wu J. C. Wu S. M. (2013). Induced pluripotent stem cell-derived cardiomyocytes for cardiovascular disease modeling and drug screening. Stem Cell Res. Ther. 4, 150. 10.1186/scrt380 24476344 PMC4056681

[B51] Sopko N. Qin Y. Finan A. Dadabayev A. Chigurupati S. Qin J.. (2011). Significance of thymosin beta4 and implication of PINCH-1-ILK-alpha-parvin (PIP) complex in human dilated cardiomyopathy. PLoS ONE 6:e20184. 10.1371/journal.pone.0020184 21625516 PMC3098280

[B52] Tsukada T. Kotlyanskaya L. Huynh R. Desai B. Novak S. M. Kajava A. V.. (2011). Identification of residues within tropomodulin-1 responsible for its localization at the pointed ends of the actin filaments in cardiac myocytes. J. Biol. Chem. 286, 2194–2204. 10.1074/jbc.M110.186924 21078668 PMC3023515

[B53] van Berlo J. H. Maillet M. Molkentin J. D. (2013). Signaling effectors underlying pathologic growth and remodeling of the heart. J. Clin. Invest. 123, 37–45. 10.1172/JCI62839 23281408 PMC3533272

[B54] Vatta M. Mohapatra B. Jimenez S. Sanchez X. Faulkner G. Perles Z.. (2003). Mutations in Cypher/ZASP in patients with dilated cardiomyopathy and left ventricular non-compaction. J. Am. Coll. Cardiol. 42, 2014–2027. 10.1016/j.jacc.2003.10.021 14662268

[B55] Wang R. Kaul A. Sperry A. O. (2010). TLRR (lrrc67) interacts with PP1 and is associated with a cytoskeletal complex in the testis. Biol. Cell 102, 173–189. 10.1042/BC20090091 19886865 PMC2952927

[B56] White D. E. Coutu P. Shi Y. F. Tardif J. C. Nattel S. St Arnaud R.. (2006). Targeted ablation of ILK from the murine heart results in dilated cardiomyopathy and spontaneous heart failure. Genes Dev. 20, 2355–2360. 10.1101/gad.1458906 16951252 PMC1560410

[B57] Will R. D. Eden M. Just S. Hansen A. Eder A. Frank D.. (2010). Myomasp/LRRC39, a heart- and muscle-specific protein, is a novel component of the sarcomeric M-band and is involved in stretch sensing. Circ. Res. 107, 1253–1264. 10.1161/CIRCRESAHA.110.222372 20847312

[B58] Yan J. Aldrich R. W. (2010). LRRC26 auxiliary protein allows BK channel activation at resting voltage without calcium. Nature 466, 513–516. 10.1038/nature09162 20613726

[B59] Yang C. Zeng X. H. Zhou Y. Xia X. M. Lingle C. J. (2011). LRRC52 (leucine-rich-repeat-containing protein 52), a testis-specific auxiliary subunit of the alkalization-activated Slo3 channel. Proc. Natl. Acad. Sci. U.S.A. 108, 19419–19424. 10.1073/pnas.1111104108 22084117 PMC3228475

[B60] Zemljic-Harpf A. E. Miller J. C. Henderson S. A. Wright A. T. Manso A. M. Elsherif L.. (2007). Cardiac-myocyte-specific excision of the vinculin gene disrupts cellular junctions, causing sudden death or dilated cardiomyopathy. Mol. Cell. Biol. 27, 7522–7537. 10.1128/MCB.00728-07 17785437 PMC2169049

[B61] Zhang J. Yan J. (2014). Regulation of BK channels by auxiliary gamma subunits. Front. Physiol. 5:401. 10.3389/fphys.2014.00401 25360119 PMC4197896

[B62] Zheng M. Cheng H. Li X. Zhang J. Cui L. Ouyang K.. (2009). Cardiac-specific ablation of Cypher leads to a severe form of dilated cardiomyopathy with premature death. Hum. Mol. Genet. 18, 701–713. 10.1093/hmg/ddn400 19028670 PMC2722217

